# Perceptions and Representations of Senior Nursing Students about the Transition to Professional Life during the COVID-19 Pandemic

**DOI:** 10.3390/ijerph19084466

**Published:** 2022-04-07

**Authors:** Cidália Castro, Ricardo Antunes, Júlio Belo Fernandes, João Reisinho, Rita Rodrigues, João Sardinha, Célia Vaz, Luís Miranda, Aida Simões

**Affiliations:** 1 Escola Superior de Saúde Egas Moniz, Caparica, 2829-511 Almada, Portugal; cidaliamscastro@gmail.com (C.C.); riantunes2003@sapo.pt (R.A.); celiatavaresvaz@gmail.com (C.V.); luispintomiranda@hotmail.com (L.M.); aidasimoes@gmail.com (A.S.); 2 Centro de Investigação Interdisciplinar Egas Moniz (CiiEM), Caparica, 2829-511 Almada, Portugal; 3 Grupo de Patologia Médica, Nutrição e Exercício Clínico (PaMNEC)—Centro de Investigação Interdisciplinar Egas Moniz (CiiEM), 2829-511 Almada, Portugal; 4 Centro Hospitalar Barreiro Montijo, 2830-003 Barreiro, Portugal; jprhrh@gmail.com (J.R.); ritasrodriigues@gmail.com (R.R.); joaocsardinha@hotmail.com (J.S.)

**Keywords:** students, nursing, perception, student experience, transition to professional life, COVID-19

## Abstract

The COVID-19 pandemic caused disruptions in education systems worldwide. The suspension of face-to-face lectures and clinical placements directly impacted nursing students’ learning. This study aimed to identify the perceptions and representations of senior nursing students about the transition to professional life during the COVID-19 pandemic. This descriptive, observational, cross-sectional study used a web-based survey from a convenience sample of 162 senior nursing students, from nine different nursing schools. Data collection was carried out in the second quarter of 2020. Male students have more negative representations related to training (*p* = 0.048); working students have a better perspective of professional integration (*p* = 0.038); students who are in a relationship have a more positive perception of interaction with patients (*p* = 0.047); those who have already defined a service of choice have less insecurity and less fear of making mistakes (*p* = 0.043). Those who report anxiety about their first place of work have more negative representations about the future in other professional dimensions. The COVID-19 pandemic represents a frequent concern among students. However, it is a dimension that does not negatively contaminate other representations about the professional future. Overall, students showed concerns regarding their performance in providing direct care to the patient and lived up to their fellow nurses’ expectations.

## 1. Introduction

A novel coronavirus from patients with pneumonia in Wuhan, China, in December 2019, marked the beginning of the pandemic known as COVID-19 [1]. In this overall context, nurses have been positively recognized as key actors in the management of this public health crisis, and it is equally clear that the pandemic has had a severe impact on nursing care [2]. However, fears associated with this pandemic are not all distributed in the same way and have touched some individuals and communities more than others [3]. In the nursing field, it is not only in health professionals that the impact of the pandemic is felt. It is also essential to understand how this situation has impacted nursing students. 

In March 2019, the World Health Organization characterized this infection as a pandemic [4]. This situation has generated significant changes in the functioning of higher education institutions. In this context, the Portuguese government decreed a set of exceptional and temporary measures to prevent the spread of the infection, with the suspension of classroom teaching activities being one of them [5]. The United Nations Organization for Culture, Science, and Education estimates that about 70% of the world’s student community has been affected by this measure [6].

The nursing degree combines theory and practice, clinical placement being a mandatory part of education to become a registered nurse. It allows the integration of theory and practice to attain the core competencies for admittance to the register [7]. Clinical placement is fundamental in nursing academic training, regarding the consolidation of theoretical–practical knowledge and skills, culminating the last clinical placement with the transition to professional life [8].

The concerns of nursing students are mainly based on the differences observed between clinical placement and professional clinical practice, causing senior students to undergo an early socialization phase, in which they idealize their profession based on their learning experiences [8,9]. Moreover, due to the pandemic situation, all face-to-face classes were replaced by teaching through digital platforms [10]. Given the social confinement and restrictions on people’s movement, health institutions temporarily suspended the clinical placement [10]. This scenario caused great uncertainty about continuing and completing the courses, especially for senior nursing students in their transition to professional life. 

Recent studies have identified mental health problems and sleep disturbances among nursing students associated with the COVID-19 pandemic. A systematic review of seventeen studies, representing 13,247 nursing students, showed that the health problems with the highest prevalence in nursing students were depression (52%), anxiety (32%), and stress (30%), followed by sleep disturbances (27%) [11].

Another study exploring the clinical placement experiences of nursing students during the COVID-19 pandemic concluded that students need to learn how to lower their fear and self-manage the emotional burden to be a caring presence for the patients who are suffering from the disease and isolation [12].

The transition from student to health care professional is challenging due to the inexperience and the continuous need for learning that can result in insecurity and fear [13,14]. During this period, newly graduated nurses decide to commit to or abandon the profession [14,15].

The COVID-19 pandemic profoundly impacted the healthcare sector, generating unprecedented learning opportunities for students, yet the pandemic also may endanger their learning trajectory [16]. In this way, the present investigation has the central objective of identifying the perceptions and representations of senior nursing students about the transition to professional life during the COVID-19 pandemic.

## 2. Materials and Methods

### 2.1. Study Design and Participants

Based on the objectives of the investigation, we developed a descriptive, observational, and cross-sectional study with a mixed approach, in a sample of final year students in the nursing degree course, from nine higher education institutions in the region of Lisbon and Alentejo, in Portugal. We used a non-probabilistic convenience sample of 162 students. In order to analyze regional differences, schools were grouped into two dimensions according to locations: the Lisbon context, which represents the urban and cosmopolitan dimension, and Alentejo, which represents a more inland and rural context.

### 2.2. Procedures

An online pre-test was first applied to a group of 15 senior nursing students. Participants were questioned to understand their perceptions about the survey. They considered the instrument sufficiently clear, objective, and comprehensive and did not present questions that could be ambiguous or equivocal. The pre-test allowed researchers to conclude that the survey was suitable for this study. 

Data were collected in the second quarter of 2020 by applying an online survey submitted in Google forms. Study participants were contacted through social networks and invited to participate through a link to access the survey. 

### 2.3. Instruments

The database was structured in three dimensions:

(i). Sociodemographic and academic attributes: sex, age, marital status/relationships, nursing school location, and student worker status;
(ii). Concerns and difficulties in this transition period: here, the students rated their perceptions on a 4-point scale with statements ranging from 1 = totally disagree, 2 = disagree, 3 = agree, and 4 = totally agree. In addition to these, an open-ended question was included asking: “What difficulties do you think you will experience in the transition from higher education to professional life?”;
(iii). Choice and idealization of workplaces after completing the course. This third dimension was based on two open-ended questions. The first wanted to know if the students already had a health service in mind where they would like to work when they finished the course. A second question wanted to know which service was elected.

### 2.4. Analysis

Exploratory and inferential analyses were carried out. Data were organized and classified using statistical treatment based on the SPSS software for Windows, version 26.0. 

Firstly, descriptive statistics were calculated. Open-ended answers were analyzed according to Braun, Clarke, Hayfield, and Terry’s [17] method, which involved four phases: pre-analysis, encoding, categorization, and interpretation of data. This process was performed by two researchers independently. They assigned codes to the meaning units using the participants’ own words. Next, categories and subcategories were developed inductively from the initial codes, based on the differences and similarities in the participants’ answers. Afterward, two other researchers reviewed the participant quotes and matched each quote to one of the identified categories. Finally, the whole research team reviewed the final findings. Nine categories emerged from the analysis. In a second phase, the information from a set of rating scale variables was summarized in orthogonal indices through a principal component analysis (PCA). In this process, the initial 25 variables were reduced to 15. The application of PCA allowed the extraction of 4 components via a varimax rotation. The suitability of the process was proven using Bartlett’s test of sphericity (x^2^ (102) = 876.435 with a *p* < 0.001) and the Kaiser–Meyer–Olkin statistical test (KMO = 0.746; acceptable value > 0.5) [18]. The eigenvalue rule > 1 and the graphical visualization of the scree plot was used as a component retention criterion. The extraction of these four components explained 64.5% of the total variance. The internal consistency of each component was measured using Cronbach’s alpha (accepting values > 0.6). Finally, the arithmetic mean was calculated for each of the new four index variables, based on the highest loadings in each component.

Thirdly, an inferential statistics analysis was carried out. To determine which tests would be used, the normal distribution was analyzed through the Kolmogorov–Smirnov test and the homogeneity of variances through the Levene test. The dataset was not correlated with normal distribution but the homogeneity of the variances was verified, so non-parametric tests were used. The Mann–Whitney U-Test was used in situations where independent variables were only two. When the independent variables were more than two, the Kruskal–Wallis test was used. A *p*-value lower than 0.05 was considered statistically significant.

Fourthly, a multiple correspondence analysis (MCA) was carried out. The set of input variables was selected through a process of analyses that included different solutions in introducing variables and in the grouping and composing categories. The definition of the number of dimensions resulted from graphic (scree plot) and statistical analyses of the eigenvalues of the first five dimensions. The first two dimensions were selected based on values that stood out from the others. The designation of the dimensions was based on the interpretation of the categories of the variables, using their coordinates, the contributions of the categories to the inertia of the dimensions and, finally, the quality of the representation of the categories to inertia of point.

### 2.5. Ethics and Procedures

Before conducting the study, a research protocol was analyzed and approved by the Egas Moniz Higher School of Health Board of Directors and the Institutional Ethics Committee (Date: 5 May 2020; ID 888/2020).

On the first page of the survey, it was stated that participation was entirely voluntary, and participants could decline to answer any questions. Participants were also free to change or review their responses or voluntarily quit at any time. Implied consent was obtained when the participants proceeded to the next page.

## 3. Results

A total of 162 nursing students completed the online survey. As shown, in this sample (Table 1), most respondents are female (87.0%). The mean age is 23 and SD = 3.85 years. It should be noted that since the target population consists of students who are all finalists, the age does not vary much; 82% are between 21 and 23 years old. In this sense, age will not be considered a significant parameter in this study. 

In this study, 85.2% of the students are from nursing schools located in urban areas around Lisbon (33%), and 14.8% come from schools located in Alentejo.

Most students identify themselves as single (84.4%), and only a minority express that they are in a relationship (15.6%); a small group consists of student-workers (12.2%).

Regarding the expression of emotions, most students report anxiety associated with their first workplace (62.7%). However, 57.3% have already defined a service they would like to work. About the elected services chosen for the beginning of their nursing careers, the majority of students (86.1%) intend to work in a hospital setting. In this context, the elected workplaces are constituted as services with greater technological incorporation, such as “emergency services + intensive care units” (25.7%), followed by services with a higher degree of medical specialization, such as “obstetrics + pediatrics” (23.8%), “surgeries” (18.8%) and, finally, services with less incorporation of technology, such as “medicines + psychiatry” (17.8%). Lastly, outside the hospital context, “primary health care”, together with “end of life and palliative health care” (13.9%), are the least attractive types of services for senior nursing students.

### 3.1. Concerns during the COVID-19 Pandemic

Nine categories (Table 2) emerged from the content analysis, with respect to participants’ concerns regarding the transition to professional life during the COVID-19 pandemic.

The category completing the course refers to difficulties in finishing the course, in terms of theoretical and practical assessments. It represents a concern with little expression at this stage (5.2%).

The category application of knowledge represents the most frequent concern (20.1%). It reflects the difficulties in articulating theoretical knowledge in future professional practice contexts, especially in areas such as safe medication administration and communication with the multidisciplinary team.

The COVID-19 category, the second most frequent (18.7%), encompasses diverse concerns, ranging from the more general social risks and fears of the unknown to the more concrete situations related to class interruptions that can postpone the course completion. 

The category labor market and perspectives reflects a theme that does not constitute a central apprehension; only 3.7% of students expressed concerns about career progression, remuneration, working conditions, or the possibility of having difficulties in finding work after the end of the course. 

The category practice deficit (7.5%) mainly reflects the lack of practical skills to manage health devices and technologies.

A more frequent concern (13.4%) refers to the category field of nursing practice. It reflects, above all, the situations of students who have already chosen a unit or institution to work. Therefore, they do not see themselves working in another area of care.

Fear of making mistakes, the third most frequent concern (14.2%), reflects the inexperience associated with a lack of confidence and the consequent fear of making mistakes that could harm patients in the professional context.

Meeting Expectations, with 9.2%, reflects expectations in the ability to respond to challenges in the nursing team, associated with knowledge and technical skills as a recent health professional, and the responsibility related to the social role of nurses in society.

The introduction period (8.2%) concerns the ways and possibilities of stress management in the first job and how they will be received as newly graduated nurses in highly complex care contexts.

### 3.2. Principal Component Analysis

A PCA was performed on a set of 15 scale variables to reduce the number of variables and simultaneously identify composite dimensions. This allowed the extraction of four dimensions. Table 3 shows the final PCA result, with the loading factor values after rotation.

The analysis of the information in Table 4 allows us to differentiate students’ perceptions around four factors of concern: (i) concerns related to lack of knowledge; (ii) concerns related to patient interaction; (iii) concerns related to professional integration and, (iv) concerns related to insecurity and health error.

The composite variables range from 1 to 4; here, lower scores reflect more positive perceptions regarding the transition to the professional field, while higher values represent more negative perceptions. In this analysis, as we see in Table 3, the most positive dimension refers to concerns related to patient interaction (2.1 ± 0.549). Secondly, the dimension concerns related to lack of knowledge, which is mainly linked to the difficulties in articulating theoretical knowledge in practical contexts (2.7 ± 0.528), is slightly situated in the field of negative perceptions, if we consider that the neutral value is represented by 2.5. The other two dimensions are more clearly situated at the pole of the most negative representations: the dimension concerns related to professional integration (3.4 ± 0.481) and, finally, concerns related to insecurity and health error (3.8 ± 0.333). 

Next, we analyzed a set of independent variables about the individual attributes of students and their choices in articulation with these four dimensions (Table 5). 

As shown in Table 5, regarding sex, male students mainly present higher averages in the dimensions under analysis, which show more negative representations than female students, except for the dimension related to “concerns related to professional integration”. The greatest differentiation in sex, which is statistically significant (*p* = 0.048), occurs in the dimension related to academic training, in which male students, compared to female students, have more concerns related to lack of knowledge.

The regional dimension linked to the location of nursing schools, whether in an urban context, such as Lisbon, or a more rural one, such as Alentejo, does not appear in this study as a differentiating factor in perceptions and representations of the professional future among senior nursing students.

On the other hand, being in a relationship appears as a factor that favors positive representations in all dimensions, highlighting better prospects in concerns related to patient interaction (1.88 ± 0.44; *p* < 0.05). In this same sense, an attribute that appears with more positive representations relates to student-workers, who presented lower averages in all four dimensions. The most significant differences, where student-workers have more positive representations, are in the perception of future, with fewer concerns related to professional integration (2.47 ± 0.49; *p* < 0.05), and also lower concerns related to insecurity and health error (3.56 ± 0,47; *p* < 0.05).

Regarding emotions, students who report greater anxiety about their first workplace also have more negative perceptions in the four dimensions. We highlight, as statically significant, the intersections with the dimension concerns related to patient interaction (*p* = 0.006) and the dimension of concerns related to insecurity and health error (*p* = 0.044).

The next crossover shows, in general, that students who already have a clearer definition of the service they would like to work in also have more positive representations. This appears in three of the four dimensions analyzed. Although not statistically significant, the only dimension in which they present more negative results is concerns related to insecurity and health error (*p* = 0.192). On the other hand, the statistically significant result appears at the intersection with the dimension of Concerns related to professional integration (*p* = 0.047), which means that those who already have a chosen service have fewer apprehensions about their future professional integration. 

Regarding elected health services, there are no statistically significant differences. However, services with greater technological incorporation and medical specialization have lower average values, which means they are associated with more positive representations.

### 3.3. The Social Space of Student Representations in the Professional Field

A final statistical analysis considered the complexity that characterizes these phenomena related to social representations, which implies a multidimensional approach, where different factors interact. This is an empirical research situation, in which it is required to interpret the meaning of the whole, but whose complex configuration requires a multifaceted and relational approach to the object of study. As in other studies [19], to operationalize this multidimensional structure, an MCA was carried out. Table 6 presents the final set of variables that allowed for a multidimensional characterization of a structured space of students’ perceptions and representations regarding their professional future.

**Figure 1:**
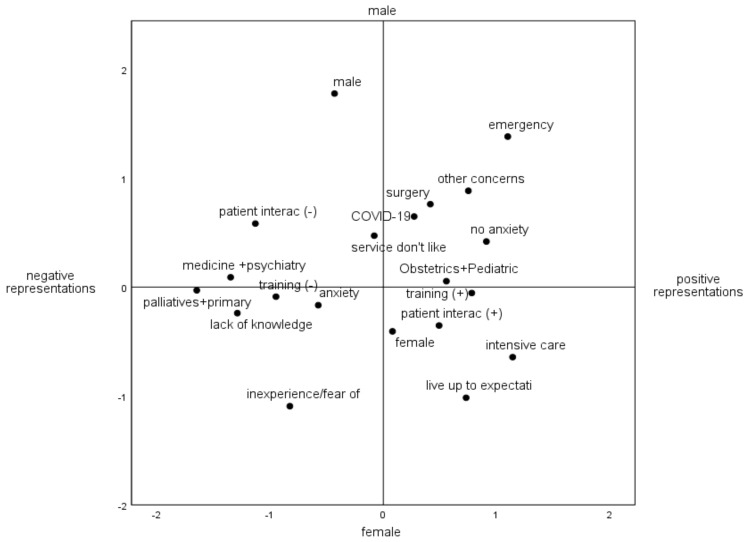
Representations of nursing students in the period of transition to the profession.

The analysis of the topographic configuration, translated in Figure 1, was anchored in two-dimensional logic. The first dimension, oriented along the horizontal axis, is structured according to different degrees of perceptions about the professional future, grouping the most negative representations on the left side, in contrast to the more positive on the right side. The second dimension corresponds to the vertical axis that differentiates this social space by sex.

Regarding the first dimension, the visualization of the information along the horizontal axis allows us to perceive that the categories that demonstrate more negative perceptions are concentrated on the left side. For example, we can see a more negative representation related to the nursing course in the category “Training (-)”, which, in turn, is closer to greater difficulties in articulating theoretical knowledge in practice. In addition, these categories are in close proximity to feelings of lack of autonomy, inexperience, and fear of making mistakes, in the context of upcoming work. Further, there are greater concerns about patient interaction in these left-side quadrants and the expression of more anxiety regarding the first workplace.

Along this horizontal axis, there is a decrease in negative representations to the other extreme, on the right side of the figure, where categories, in general, concentrated more positive perceptions in the domains of academic training, health care, and interaction with the patient, and also revealing expression of less anxiety concerning the first workplace.

The workplaces chosen by the students were grouped according to the criteria of technological incorporation and degree of specialization. Thus, in the left quadrants, we can see services with less technological incorporation, such as palliative care grouped with primary health care, and medicine wards grouped with psychiatry. The visualization of this type of service, with less technological incorporation, allows us to perceive that they are in the quadrants close to the categories with the most negative representations. On the other hand, services with a higher degree of technological incorporation, such as intensive care units (ICU), grouped with the operating room and emergency services, as well as more specialized services, such as surgery, obstetrics, and pediatrics, are all located in quadrants close to categories with more positive representations. The expression of concerns related to the COVID-19 pandemic appears in a more central position in this figure, which means that it is a concern reported by many students. However, the concerns with COVID-19 are located closer to categories that denote less negative perceptions. 

The crossing of this axis, of perceptions and social representations, with sex, allows for another type of differentiation. It is important to mention that sex analysis in this study was not a significant driving force in differentiating representations in this group of senior students. However, it should be noted, as shown in Figure 1, that male students have slightly more negative perceptions about the dimension of training than female students. On the other hand, the female sex presents greater differentiation of representations, but above all, it is around the feminine space that expressions of the greatest fears emerge. This can also mean a greater retraction of male students in self-assessment negatively. For example, it is mainly in the female population that, and in a more pronounced way, the concerns and fears about meeting the expectations of future colleagues in the first work contexts are concentrated.

## 4. Discussion

The transition from student to health care professional is challenging due to inexperience and the continuous need for learning that can result in insecurity and fear [13,14]. During this period, newly graduated nurses decide to commit to or abandon the profession [14,15].

The COVID-19 pandemic profoundly impacted the healthcare sector, generating unprecedented learning opportunities for students, yet the pandemic may also endanger their learning trajectory [16].

Exploring the impact of the COVID-19 pandemic on senior nursing students may influence the educational practices and enable schools to help students overcome this specific transition.

The present study describes how the representations of senior nursing students were affected by the COVID-19 pandemic. The participants in this study, who now leave their student life and project themselves as professionals, experience a moment of natural uncertainty in the context of highly professionalized and structured work, as health institutions are currently constituted.

This study shows that the participants’ main concerns are related to insecurity towards the patient and insecurity towards health organizations. In these two levels of analysis, the micro and the organizational level, the main concerns of students relapse. In this sense, students’ concerns do not have much to do with academic training and the acquisition of theoretical knowledge. In the same sense, it is also clear that concerns do not fall on employability and labor market issues.

It is important to emphasize that at the micro-level of analysis, in the interaction with the patient, the relational and communicational skills in face-to-face interaction with patients or their families are not a factor of great concern at this moment of transition. The information contained in the variables allows us to conclude that face-to-face interaction with the patient, communication with the family, and the possible diversity of patients’ attributes do not cause great apprehensions or insecurities in this period of transition. The main problem is, above all, in the interaction with the patient, the fear of making serious mistakes. Although this fear arises predominantly, it appears that there is no direct connection with the lack of theoretical–practical knowledge. 

The fear of making mistakes has to do with the issues of still feeling insecure, with little experience in practical contexts of action and provision of health care, but it is, above all, how this insecurity can lead them to make mistakes that could harm patients. Several authors refer that the transition from student to a healthcare professional is challenging [9,13,14]. As in previous studies, senior nursing students reported uncertainties about the future. They feel that they cannot always perform the nurse’s role, nor can they even find themselves capable of taking responsibility for caring for patients due to lack of skills, knowledge, or simply due to the fear of making mistakes [20,21]. The transition from student to registered nurse can be difficult due to changes in roles and expectations. To become a competent nurse, the nature of a senior nursing student’s transition plays an essential role. Students need to adjust to the fact that they will no longer perform procedures and activities with direct supervision in a controlled environment to practice independently as registered nurses [22].

Professional integration emerges as a central concern at an intermediate analytical level, which we can place in organizational contexts. The study findings reflect the students’ apprehension about incorporation and socialization in work contexts and multidisciplinary health teams. A central concern among students is how future peers will assess them as nurses. It seems to be very important to be positively evaluated by peers. Living up to expectations, being able to respond to problems, as a member considered an integral part of health teams, and the fear of exclusion for being recent professionals constitute a set of main concerns.

Being a nurse can be challenging and unpredictable because of the workload, complexity of health problems faced by patients and the healthcare environment [23]. To be able to provide high-quality care, teamwork is needed. In the transition to become nurses, students experience anxiety and low self-confidence concerning their knowledge and skills. To facilitate this transition, newly graduated nurses need to be supported within the work environment [24]. Work colleagues have a significant role in ensuring the newly graduated nurses successfully transition into practice as they offer guidance to new graduates [25]. Besides formal orientation and mentorship, the support from other newly graduated nurses can facilitate their role transition [26]. Nursing education has limits in preparing students for every single clinical situation. Therefore, healthcare institutions should follow the European Federation of Nurses Associations [27] and International Council of Nurses [28] guidelines and facilitate a structured introduction to newly graduated nurses. The introduction period is fundamental for the continuous development of newly graduated nurses and should be led by experienced nurses. By following these guidelines, healthcare institutions enable newly graduated nurses to build relationships with other professionals and acquire more confidence in complex nursing scenarios, such as emergencies and drug administration [29].

Regarding a macro level of analysis, related to a broader social context, and the extrinsic reward values of the profession, such as qualification prestige, good remuneration, working conditions, and career progression, all these are dimensions that did not give rise to negative representations among students. However, at this macro level, the most frequent concern was associated with the COVID-19 pandemic.

The research results showed a tendency for the overlapping of negative representations. For example, students with negative representations about academic training also present more negative representations about the interaction with the patient and with all other dimensions analyzed. Furthermore, they are the ones who most reveal higher levels of anxiety related to the first place of work. Regarding the context of the pandemic, it is essential to emphasize that these data seem to confirm that the pandemic situation, despite occurring as a frequent concern, does not appear to negatively contaminate the students’ perceptions about the other dimensions. Undoubtedly, the COVID-19 pandemic is an essential element in how students evaluate their future. However, it appears as an external factor, with a more neutral intensity and without the great capacity to negatively affect students’ representations in this transition period.

From another perspective, it was possible to identify factors that contribute to more positive representations. In this sense, the following stand out: working students, those who are married or in a relationship, and students who have already defined, more clearly, the health service where they would like to work. In these situations, COVID-19 does not seem to emerge as an element of breach of trust.

In the domain of health service choices, as in previous studies, the hospital continues to be a highly attractive pole [30,31]. There is a clear trend, in which services with greater technological incorporation and greater medical specialization represent a professional domain of greater attraction for students [32,33]. In the same tendency, students who elected the services with the greatest specialization and technological incorporation also have the most positive representations in this transition period.

### Study Strengths and Limitations

Rigorous web-based surveys can be valuable for producing evidence faster and more cheaply than more traditional approaches [34]. 

Given the paucity of studies exploring and understanding the impact of the COVID-19 pandemic on senior nursing students, this study presents itself as one of the first in this field. 

We consider that this study has some limitations, especially the small sample size. The disproportion that normally exists between male and female students in nursing requires a larger sample. On the other hand, it would be interesting to compare the representations of senior nursing students with students from different years. In addition, as identified in previous studies reliant on data collection from online surveys, selective participation could be a particular limitation due to the automatic exclusion of potential participants who do not use social media platforms. Another limitation of this study is that the open-ended question responses could not be explored with immediate follow-up questions that would potentially deepen the knowledge on the subject. Therefore, more research is needed to better understand this subject.

## 5. Conclusions

This research offers an overview of senior nursing students’ perceptions and representations regarding the transition to professional life during the COVID-19 pandemic. It is possible to assume that senior nursing students feel uncomfortable with the transition from student to registered nurse.

Students direct much of their concerns to what we could call a humanistic scope. They are more concerned with their performance in providing direct care to the patient and avoiding errors. Issues related to work dimensions, such as low pay, difficulties in career progression, or poor working conditions, are rare in this study. The main concern is around demonstrating a good performance with colleagues, the fear, insecurity, inexperience, and not affecting or harming patients. 

Reading together the four various composites and the other variables allows us to highlight the concerns of the students centered on humanist ideals. They show concerns about providing good care, avoiding errors, and being at the level of what is expected of a nurse. The knowledge acquired in the course does not seem to be an important obstacle in the first encounter with work. There is a tendency that can be seen in the different variables, which is the need to live up to expectations and apply the acquired knowledge well, which is also reflected in the concern for integration in services. It conveys the perception that integration is a crucial phase for relieving anxiety.

The findings of this research illuminated key areas that educators must focus on to support students in the transition to professional life. Strategies must be developed to help students lower their fear and anxiety and support them to manage the emotional burden associated with this transition. By preparing students to manage the emotional burden, nursing educators can significantly impact students’ successful transition to practice. 

Healthcare institutions also have the responsibility to facilitate the successful transition of newly graduated nurses. Various strategies, such as the European Federation of Nurses Associations and International Council of Nurses guidelines, should be put in place to facilitate a structured introduction for newly graduated nurses.

With COVID-19 cases rising worldwide at worrying rates, it is essential to understand and address the concerns felt by students and their foreseen difficulties, to help them transition to becoming healthcare professionals. 

## Figures and Tables

**Table 1:** Participants’ characteristics.

Variables ^1^	Participants	
	Total Students *n* = 162	
	Counts (*n*)	Percentages (%)
Sex		
Male	21	13.0
Female	141	87.0
Nursing School—localization context		
Urban context—Lisbon	138	85.2
Rural context—Alentejo	24	14.8
Relationships		
Being in a relationship	20	15.6
Single	108	84.4
Student worker status		
Yes	19	12.2
No	137	87.8
Anxiety at first job		
Anxiety	89	62.7
Not anxious	53	37.3
Have an elected service?		
Yes	82	57.3
No	61	42.7
Elected services		
Emergency + ICU	26	25.7
Medicine + Psychiatry	18	17.8
Surgery	19	18.8
Obstetrics+ Pediatrics	24	23.8
Palliatives + Community	14	13.9

^1^ Presence of missing data.

**Table 2:** Concerns during the COVID-19 pandemic.

	Participants (*n* = 132)	
	Counts (*n*)	Percentages (%)
Categories		
Completing the course	7	5.2
Application of Knowledge	27	20.1
COVID-19	25	18.7
Labor market perspectives	5	3.7
Practice deficit	10	7.5
Field of nursing practice	18	13.4
Fear of making mistakes	19	14.2
Meeting Expectations	12	9
Introduction period	11	8.2

**Table 3:** Rotating Component Matrix of PCA.

	Components			
	Concerns Related to:			
Variables	Lack of Knowledge	Patient Interaction	Professional Integration	Insecurity/Health Error
I’m concerned about the lack of theoretical knowledge	0.854	0.156	0.073	0.036
The lack of clinical practice worries me	0.777	−0.077	0.089	0.114
I’m concerned about the fact that I do not have a technical language to correspond to the exercise of the profession	0.747	0.172	−0.036	−0.053
I’m concerned that I do not have the necessary knowledge about medication	0.746	0.027	−0.066	0.129
I’m concerned about the differences between what I learned in college and what I observe in practice	0.661	0.235	0.106	0.073
I worry about having to talk to the patient about their suffering	0.237	0.776	−0.106	−0.02
I’m concerned that I am moved by the patient’s emotions	0.059	0.746	0.067	0.064
I’m concerned that I may be with a patient whose political, cultural, moral, and religious options are different from mine	−0.066	0.741	0.067	−0.027
I would feel uncomfortable clarifying the patient’s clinical situation to the family	0.226	0.666	0.133	0.029
It would affect me if I didn’t have a good relationship with the multidisciplinary team	−0.042	−0.026	0.887	0.11
It would affect me if I didn’t feel part of the work team	−0.041	−0.037	0.835	0.261
I’m concerned that I will be judged by other professionals	0.229	0.325	0.651	0.058
I would feel very bad if I harmed the patient	0.061	−0.022	0.135	0.915
I would feel very bad if I made a therapeutic error	0.082	−0.02	0.101	0.867
I would feel bad if I couldn’t take care of all the patients	0.138	0.148	0.385	0.531
Explained variance of each component	26.3%	17.1%	12.9%	8.2%

**Table 4:** Dimensions regarding the concerns of nursing students in the transition to professional life.

	Mean ± SD	* α	n° of Itens
Concerns related to lack of knowledge	2.7 ± 0.528	0.830	5
Concerns related to patient interaction;	2.1 ± 0.549	0.739	4
Concerns related to professional integration	3.4 ± 0.481	0.731	3
Concerns related to insecurity and health error.	3.8 ± 0.333	0.706	3

* α Cronbach’s alpha coefficient. Variables are expressed as means ± standard deviations.

**Table 5:** Mean values and standard deviations.

	Concerns Related to:							
	Lack of		Patient		Professional		Insecurity	
	Knowledge	*p* Value	Interaction	*p* Value	Integration	*p* Value	Health Error	*p* Value
Variables	Mean ± SD		Mean ± SD		Mean ± SD		Mean ± SD	
Sex (a)								
Male	3.21 ± 0.58	0.048 *	2.28 ± 0.44	0.093	3.36 ± 0.47	0.914	3.84 ± 0.31	0.24
Female	2.66 ± 0.52		2.07 ± 0.56		3.38 ± 0.48		3.75 ± 0.34	
Nursing school (a)								
Urban context—Lisbon	2.72 ± 0.64	0.415	2.09 ± 0.56	0.581	3.34 ± 0.48	0.384	3.74 ± 0.34	0.287
Rural context—Alentejo	2.81 ± 0.67		2.11 ± 0.47		3.58 ± 0.42		3.89 ± 0.25	
Relationships (a)								
Being in a relationship	2.63 ± 0.54	0.593	1.88 ± 0.44	0.047 *	3.32 ± 0.46	0.456	3.63 ± 0.38	0.095
Single	2.73 ± 0.51		2.11 ± 0.55		3.37 ± 0.48		3.77 ± 0.32	
Student worker (a)								
Yes	2.84 ± 0.67	0.427	2.01 ± 0.53	0.521	2.47 ± 0.49	0.038 *	3.56 ± 0.47	0.043 *
No	2.72 ± 0.64		2.1 ± 0.55		3.48 ± 0.48		3.79 ± 0.31	
Anxiety at first job (a)								
Anxiety	2.79 ± 0.63	0.084	2.48 ± 0.59	0.006 *	3.52 ± 0.45	0.097	3.94 ± 0.27	0.044 *
Not anxious	2.49 ± 0.63		1.81 ± 0.51		3.01 ± 0.51		3.25 ± 0.39	
Have an elected service? (a)								
Yes	2.72 ± 0.67	0.811	2.08 ± 0.57	0.617	3.18 ± 0.47	0.047 *	3.79 ± 0.31	0.192
No	2.75 ± 0.59		2.12 ± 0.51		3.66 ± 0.49		3.72 ± 0.37	
Elected services (b)								
Emergency + UCI	2.31 ± 0.48	0.288	1.81 ± 0.48	0.065	3.16 ± 0.38	0.368	3.76 ± 0.30	0.107
Medicine + Psychiatry	2.92 ± 0.71		2.30 ± 0.51		3.42 ± 0.47		3.81 ± 0.15	
Surgery	2.62 ± 0.49		2.17 ± 0.71		3.21 ± 0.48		3.75 ± 0.39	
Obstetrics + Pediatrics	2.63 ± 0.68		2.01 ± 0.46		3.20 ± 0.44		3.81 ± 0.31	
Palliatives + Primary care	2.88 ± 0.83		2.24 ± 0.67		3.38 ± 0.41		3.68 ± 0.43	

Continuous variables are expressed as means ± standard deviations. (a) Two-tailed Mann–Whitney U test; (b) Kruskal–Wallis test. * *p* values considered significant when *p* is less than 0.05.

**Table 6:** Variables and categories in the MCA.

Variables	Categories
Sex	Male
	Female
Elected services	Emergency Services
	Intensive Care Unit (ICU)
	Surgery
	Obstetrics + Pediatrics
	Medicine +Psychiatry
	Palliatives + Health Center
Concerns about the professional future	Lack of knowledge
	Inexperience/Fear of making mistakes
	Live up to expectations
	Staying in a service you don’t like
	COVID-19
	Other concerns
Anxiety related to the first workplace	Anxiety
	No Anxiety
Representations related to patient interaction	Patient interaction (+)
	Patient interaction (−)
Representations about the nursing training course	Training (+)
	Training (−)

## Data Availability

The data that support the findings of this study are available on request from the corresponding author.
